# Improvement of Sciatic Nerve Regeneration Using Laminin-Binding Human NGF-β

**DOI:** 10.1371/journal.pone.0006180

**Published:** 2009-07-09

**Authors:** Wenjie Sun, Changkai Sun, Hui Zhao, Hang Lin, Qianqian Han, Jingyu Wang, Hui Ma, Bing Chen, Zhifeng Xiao, Jianwu Dai

**Affiliations:** 1 Key laboratory of Molecular Developmental Biology, Institute of Genetics and Developmental Biology, Chinese Academy of Sciences, Beijing, China; 2 Graduate School, Chinese Academy of Sciences, Beijing, China; 3 Institute of Brain Disorders and the Key Lab for Brain Disorders of Liaoning Province, Dalian Medical University, Dalian, China; 4 Experimental Animal Center of Dalian Medical University, Dalian, China; 5 Department of Pharmaceutics, The Second Affiliated Hospital of Dalian Medical University, Dalian, China; Emory University School of Medicine, United States of America

## Abstract

**Background:**

Sciatic nerve injuries often cause partial or total loss of motor, sensory and autonomic functions due to the axon discontinuity, degeneration, and eventual death which finally result in substantial functional loss and decreased quality of life. Nerve growth factor (NGF) plays a critical role in peripheral nerve regeneration. However, the lack of efficient NGF delivery approach limits its clinical applications. We reported here by fusing with the N-terminal domain of agrin (NtA), NGF-β could target to nerve cells and improve nerve regeneration.

**Methods:**

Laminin-binding assay and sustained release assay of NGF-β fused with NtA (LBD-NGF) from laminin *in vitro* were carried out. The bioactivity of LBD-NGF on laminin *in vitro* was also measured. Using the rat sciatic nerve crush injury model, the nerve repair and functional restoration by utilizing LBD-NGF were tested.

**Findings:**

LBD-NGF could specifically bind to laminin and maintain NGF activity both *in vitro* and *in vivo*. In the rat sciatic nerve crush injury model, we found that LBD-NGF could be retained and concentrated at the nerve injury sites to promote nerve repair and enhance functional restoration following nerve damages.

**Conclusion:**

Fused with NtA, NGF-β could bind to laminin specifically. Since laminin is the major component of nerve extracellular matrix, laminin binding NGF could target to nerve cells and improve the repair of peripheral nerve injuries.

## Introduction

Sciatic nerve injuries are often caused by injections, gunshot wounds, lacerations, contusions, compressions, and iatrogenic causes [1], [2]. Injuries to sciatic nerves cause partial or total loss of motor, sensory and autonomic functions due to the axon discontinuity, degeneration, and eventual death which finally result in substantial functional loss and decreased quality of life [3], [4]. Functional deficits caused by nerve injuries can be compensated by regeneration of peripheral nerves. However, clinical and experimental evidences show that the regeneration is usually far more difficult and the results are far from satisfactory, especially after severe injuries [3], [5]. Numerous therapeutic interventions, mostly pharmacotherapeutic, have been tested to enhance functional recovery after sciatic nerve injuries. The identification of neurotrophic factors offers molecular therapy as a potential approach to enhance nerve regeneration. Among the neurotrophic factors, nerve growth factor (NGF) plays a critical role.

NGF promotes proliferation and differentiation of neurons, and also modulates the repair of injured nerves [6], [7]. It has been reported that NGF was upregulated in anticipation of the arrival of a regenerating sprout during the peripheral nerve regeneration [8]. The administration of recombinant NGF protein into injured nerves has been shown to promote nerve repair and enhance functional restoration following nerve damages [9]. However NGF simply given in solution is difficult to be retained at the injury sites because of its rapid diffusion in body fluids. Therefore, it requires periodic injection of NGF which is impractical and expensive, and excessive doses may also evoke undesirable side effects [10]–[12]. To solve these problems, many groups are working on developing NGF delivery to the nervous system via drug delivery systems [13] or transplantation of cells with/without encapsulation [14]. These systems should be improved with regard to release control, dosing, efficacy and safety.

Laminin is the ubiquitous component of the tight network of glycoproteins, collagen IV and proteoglycans in basement membranes [15]. Laminin is mainly produced by Schwann cells and widely dispersed in the peripheral nervous system (PNS) [16], [17]. After peripheral nerve injuries, laminin is significantly upregulated at the injury sites by Schwann cells and may foster axonal regeneration [18], [19]. Thus, laminin could be a suitable target for the delivery of exogenous NGF to repair PNS injuries.

Agrin is a key organizer of postsynaptic differentiation at the neuromuscular junction. The binding of agrin to laminin is required for its localization to synaptic basal lamina and other basement membranes [20]. Previous studies have demonstrated that the high-affinity interaction with the coiled-coil domain of laminin was mediated by N-terminal domain of agrin (NtA) [21]. Taking advantage of this laminin-binding domain of NtA, we produced a tripartite fusion protein to obtain laminin binding NGF-β which contains (i) the primary sequence of the mature NGF-β, (ii) NtA as a laminin-binding domain, and (iii) a 6×histidine (His) purification tag. This fusion protein was named as laminin binding domain fused NGF-β (LBD-NGF). We also produced a native NGF-β without NtA (NAT-NGF). We demonstrated that the LBD-NGF could specifically bind to laminin and maintain NGF activity both *in vitro* and *in vivo*. In the rat sciatic nerve crush injury model, LBD-NGF could target to the native nerve extracellular matrix component laminin, and could be retained and concentrated at the nerve injury sites to improve peripheral nerve regeneration.

## Results

### LBD-NGF could bind to laminin and be sustained released *in vitro*

**Figure 1:**
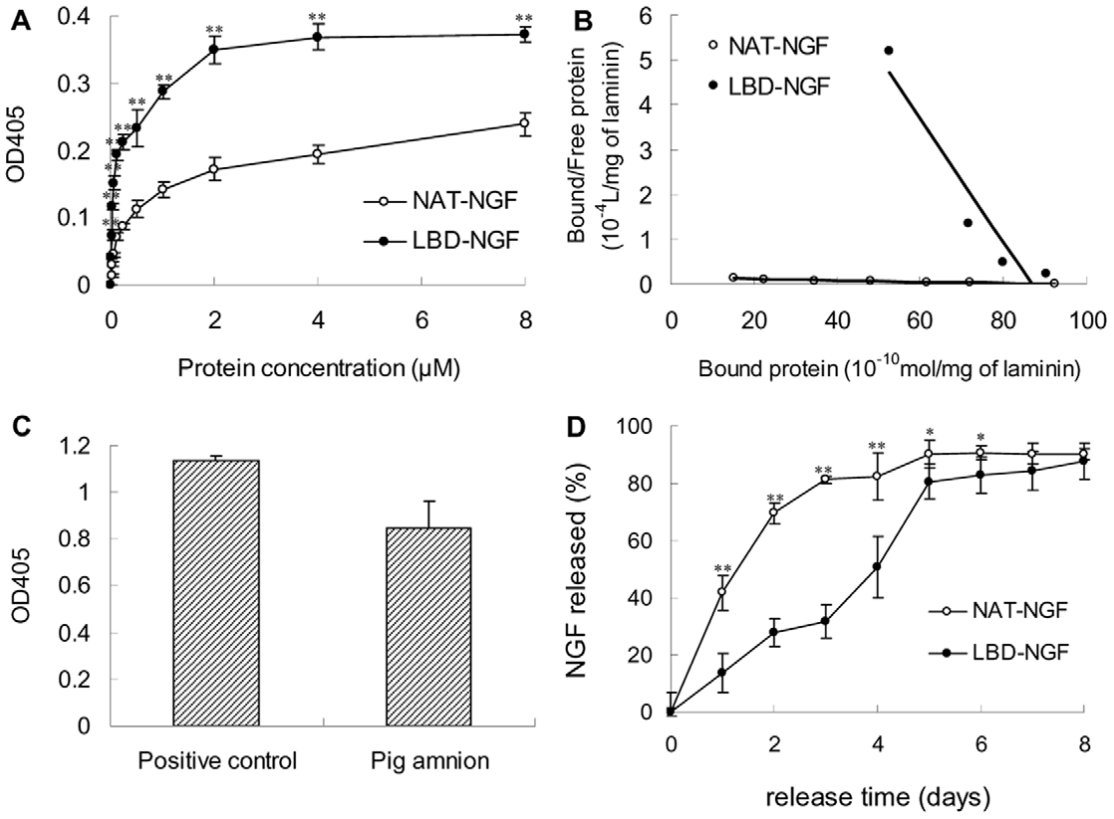
Laminin-binding and sustained release assay of NAT-NGF and LBD-NGF from laminin *in vitro*. (a) Binding curves of NAT-NGF and LBD-NGF to laminin measured by ELISA assay. (b) Kd values for laminin to NAT-NGF and LBD-NGF were calculated using Scatchard analysis. (c) Detection of laminin content in the pig amnion by ELASA assay. (d) Release curves of NAT-NGF and LBD-NGF from laminin *in vitro*. n = 6, *, P<0.05, **, P<0.01, determined by two-tailed student's *t*-test.

We measured the *in vitro* laminin-binding activities of NAT-NGF and LBD-NGF by ELISA. From the results, we found at each indicated point the OD405 of LBD-NGF was significantly higher than that of NAT-NGF, indicating that the retained LBD-NGF on laminin was more than that of NAT-NGF (n = 6, P<0.01) (Figure 1A).

Using the binding curve, we calculated the dissociation constant Kd values of NAT-NGF and LBD-NGF to laminin by Scatchard analysis. The slope of the resulting straight line equals −1/Kd (Figure 1B). The Kd value for the binding of NAT-NGF and LBD-NGF was measured as 6.25×10^−4^ M and 7.25×10^−6^ M respectively. The lower Kd value of LBD-NGF to laminin indicated that LBD-NGF bound to laminin specifically.

Using ELISA assay, we found the membrane prepared from pig amnion was rich in laminin content (Figure 1C). In the *in vitro* release experiment, sustained release of NAT-NGF and LBD-NGF was followed up to 8 days (Figure 1D). We found NAT-NGF was quickly released at the first day, whereas LBD-NGF could gradually be released from laminin. During the first 6 days, LBD-NGF retained on laminin was significantly higher than NAT-NGF (n = 6, P<0.05). These results showed that LBD-NGF could be sustained released from laminin *in vitro*.

### LBD-NGF maintained higher bioactivity on laminin *in vitro*

**Figure 2:**
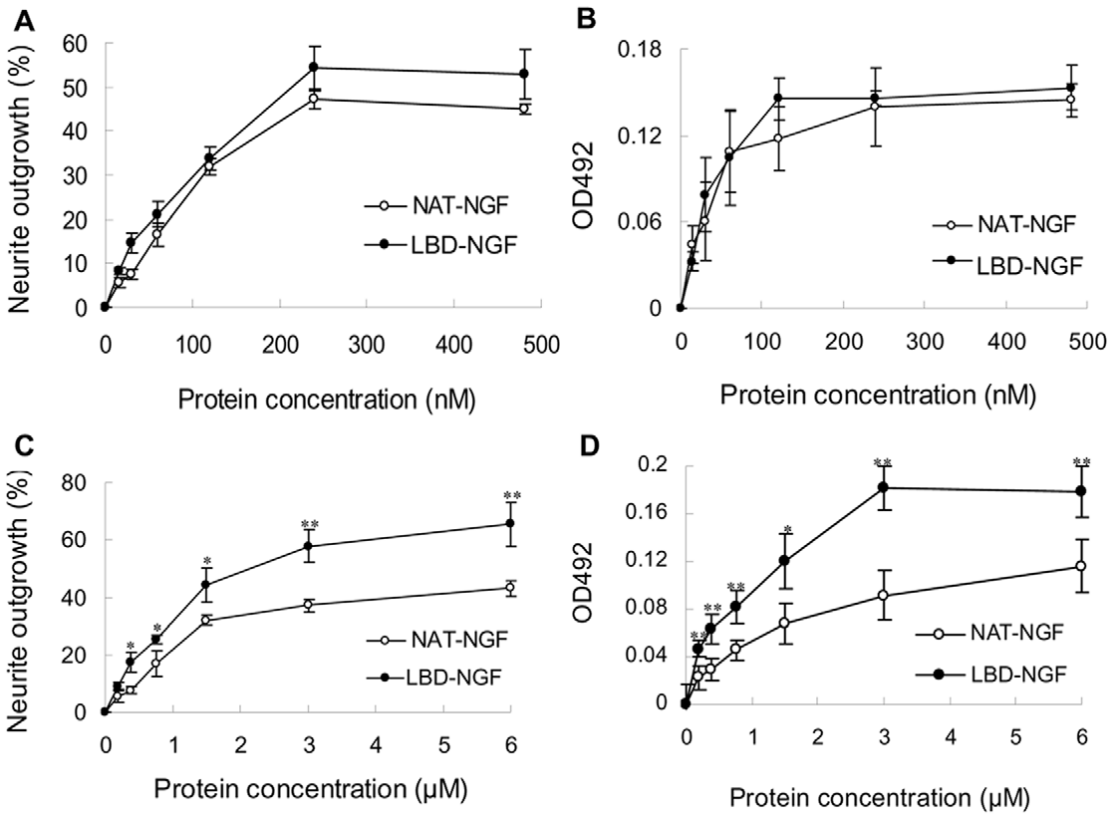
Bioactivity comparison of NAT-NGF and LBD-NGF *in vitro*. (a) Effect of NAT-NGF and LBD-NGF on neurite outgrowth in PC12 cells. (b) Effect of NAT-NGF and LBD-NGF on cell survival in PC12 cells by MTT assay. (c) Percentage of PC12 cells with neurite outgrowth on laminin stimulated by NAT-NGF and LBD-NGF. (d) PC12 cell survival on laminin stimulated by NAT-NGF and LBD-NGF was determined by MTT assay. n = 6, *, P<0.05, **, P<0.01, determined by two-tailed student's *t*-test.

We tested the bioactivities of NAT-NGF and LBD-NGF using PC 12 cell lines. Stimulated by NAT-NGF or LBD-NGF, the neurite outgrowth of PC 12 cells was observed after 24 h incubation. The percentage of cells with neurite outgrowth increased in a dose-dependent manner, but there was no significant difference among them at each concentration (Figure 2A). The application of NAT-NGF and LBD-NGF to PC12 cell lines also significantly increased cell survival. The dose-response curves of NAT-NGF and LBD-NGF were similar (Figure 2B). There was no significant difference of activity at each concentration. Thus, the bioactivity of LBD-NGF was not affected by fusing with the LBD peptide.

We then measured the bioactivity of NGF on laminin *in vitro*. The results showed at the concentration range of 0.37–6.00 µM, more cells with neurite outgrowth were founded in the LBD-NGF group than in the NAT-NGF group (n = 6, P<0.05) (Figure 2C). 3-(4,5-dimethylthiazol-2-yl)-2,5-diphenyltetrazolium bromide (MTT) assay also showed that there were more living cells in the LBD-NGF group than in NAT-NGF group (n = 6, P<0.05) (Figure 2D). These results showed that LBD-NGF maintained higher bioactivity after bound to laminin.

### LBD-NGF retained at the injury sites of sciatic nerves

**Figure 3:**
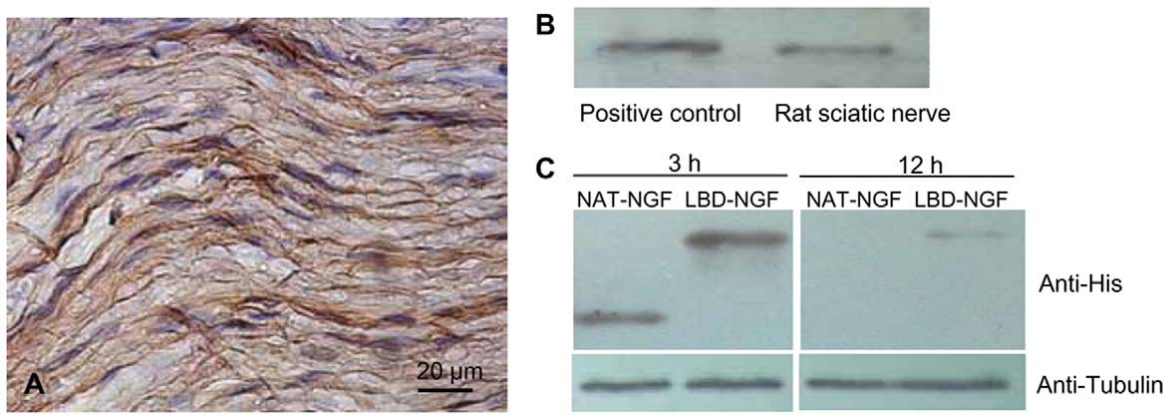
Detection of laminin content and the sustained NAT-NGF and LBD-NGF *in vivo*. (a) Immunohistochemistry of laminin in the rat sciatic nerve. (b) Detection of laminin content in the sciatic nerve by western-blotting analysis. (c) Detection of sustained NAT-NGF and LBD-NGF at the injury sites of sciatic nerves *in vivo* by western-blotting analysis.

We tested laminin content in the rat sciatic nerve. Immunohistochemistry showed the distribution of laminin in the extracellular matrix of rat sciatic nerves (Figure 3A). Using western-blotting analysis, we identified that rat sciatic nerves had rich laminin content (Figure 3B).

We evaluated whether LBD-NGF could be retained at the injury sites of sciatic nerves. Three hours and twelve hours after injection of NAT-NGF or LBD-NGF, the sciatic nerve proteins were extracted and the retained NGF was assessed by western-blotting. At 3 h after injection, the level of exogenous LBD-NGF was significantly higher than that of NAT-NGF (n = 3, P<0.05) (Figure 3C). At 12 h after injection, NAT-NGF couldn't be detected by western-blotting. However, LBD-NGF could still be detected, indicating LBD-NGF was specifically retained and enriched at the injury sites of sciatic nerves (Figure 3C).

### LBD-NGF promoted functional recovery by walking track analysis

**Figure 4:**
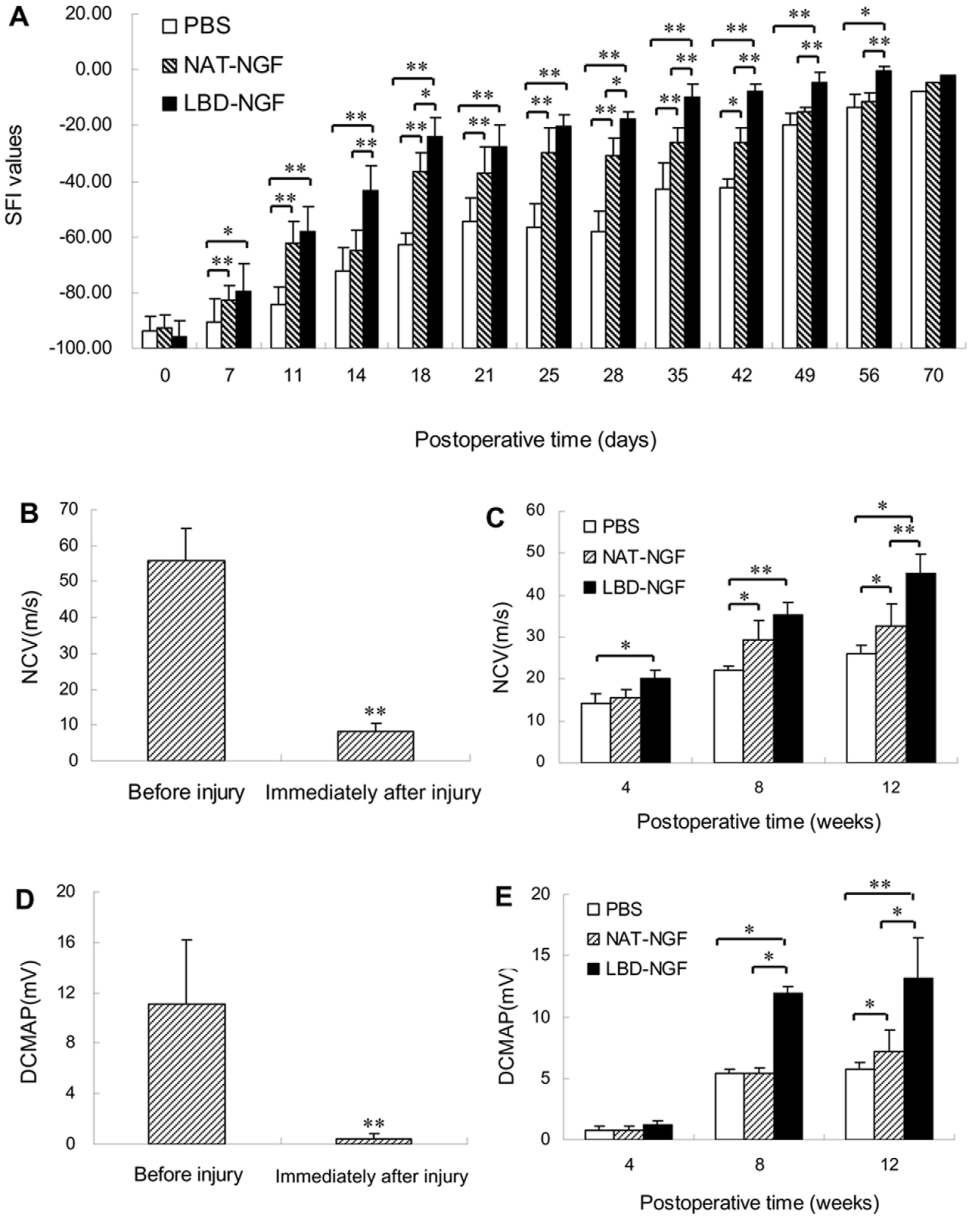
Functional recovery after sciatic nerve injury. (a) Measurements made from walking track prints were then submitted to SFI. (b) NCV evaluation before and immediately after sciatic nerve injury. (c) NCV evaluation at weeks 4, 8 12 after the sciatic nerve injury. (d) DCMAP evaluation before and immediately after sciatic nerve injury. (e) DCMAP evaluation at weeks 4, 8 12 after the sciatic nerve injury. n = 6, *, P<0.05, **, P<0.01, determined by two-tailed student's *t*-test.

At day 0 after injury, there was no difference among the groups of the control, NAT-NGF, and LBD-NGF (n = 6, P>0.05). Following several weeks after injury, the sciatic functional index (SFI) values were significantly increased in groups of NAT-NGF and LBD-NGF compared with the control (n = 6, P<0.01). At day 35, 42, 49 and 56, there were also significant differences between groups of NAT-NGF and LBD-NGF (n = 6, P<0.01) (Figure 4A).

### Electrophysiological evaluation

The results showed after injury, the nerve conduction velocity (NCV) was significantly decreased (n = 6, P<0.01) (Figure 4B), and then restored. At week 4 after injury, NCV of LBD-NGF group showed significant restoration compared with the control (n = 6, P<0.05) (Figure 4C). At week 8, NCV was significantly restored in groups of NAT-NGF (n = 6, P<0.05) and LBD-NGF (n = 6, P<0.01) compared with the control. But there was no significant difference between the groups of NAT-NGF and LBD-NGF (Fig. 4C). At week 12 after injury, there were significant differences among the three groups of the control, NAT-NGF and LBD-NGF (n = 6, P<0.05) (Figure 4C).

The distal compound muscle action potential (DCMAP) was also significantly decreased after injury (n = 6, P<0.01) (Figure 4D). At week 8, DCMAP in group of LBD-NGF was significantly restored compared with groups of NAT-NGF and the control (n = 6, P<0.05) (Figure 4E). At week 12 after injury, there were significant differences among the groups of the control, NAT-NGF and LBD-NGF (n = 6, P<0.05), and LBD-NGF showed the highest value among the three groups (Figure 4E).

### Histological analysis

**Figure 5:**
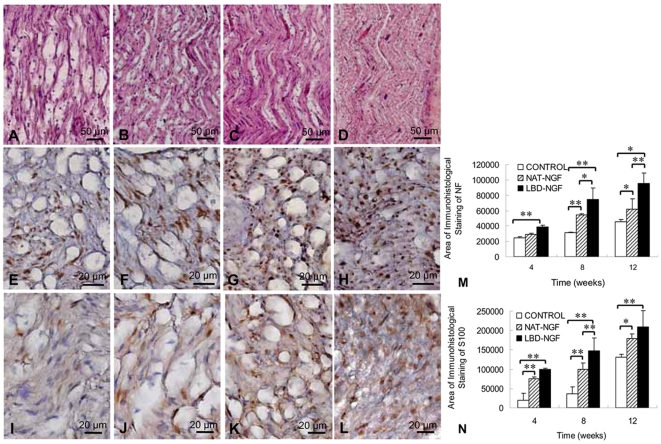
Histological analysis. (a–d) At week 8 after injury, the longitudinal sections of the control (a), NAT-NGF (b) and LBD-NGF group (c) compared with that of native nerve group (d) analyzed by H.E. staining. (e–h) At week 8 after injury, immunostaining with anti-neurofilament antibody in transverse sections of the control (e), NAT-NGF (f) and LBD-NGF group (g) compared with that of native nerve group (h). (i–l) At week 8 after injury, immunotaining of the Schwann cell marker S100 in the transverse sections of the control (i), NAT-NGF (j) and LBD-NGF group (k) compared with that of native nerve group (l). (m) The statistical analysis of neurofilament-positive area of each group. (n) The statistical analysis of S100-positive area of each group. n = 6, *, P<0.05, **, P<0.01, determined by two-tailed student's *t*-test.

The longitudinal sections at the injury sites of sciatic nerves were analyzed by hematoxylin and eosin (H.E.) staining. The results showed that at week 8 after injury, there was little nerve regeneration in the control group (Figure 5A), while significant nerve regeneration and apparent linear ordered structures were seen in the groups of NAT-NGF and LBD-NGF (Figure 5B,C). The group of LBD-NGF showed better regeneration compared with NAT-NGF.

Axonal regeneration was investigated by staining neurofilament-positive axons in transverse sections at the injury sites of sciatic nerves. Results showed that LBD-NGF promoted more axonal regeneration and nerve repair at week 8 after injury (Figure 5E–H). The statistical analysis revealed that at week 4 after injury, neurofilament-positive area in the LBD-NGF group significantly increased compared with the control (n = 6, P<0.01) (Figure 5M). At week 8 and 12 after injury, there were significant differences in neurofilament-positive area among the groups of the control, NAT-NGF and LBD-NGF, and the group of LBD-NGF had the highest value among the three groups (n = 6, P<0.05) (Figure 5M).

Immunostaining with the glia/Schwann cell marker S100 also demonstrated that LBD-NGF increased S100 expression and improved Schwann cell regeneration in response to sciatic nerve crush injury at week 8 after injury compared with the control (Figure 5I–L). The statistical analysis revealed that at week 4, S100-positive area in the LBD-NGF group significantly increased compared with the control and NAT-NGF (n = 6, P<0.01) (Figure 5N). At week 8 and 12, there were significant increases in neurofilament-positive area between the groups of NAT-NGF and LBD-NGF compared with the control (n = 6, P<0.05) (Figure 5N). There were also significant differences between the groups of NAT-NGF and LBD-NGF (n = 6, P<0.01) (Figure 5N).

### Remyelination of sciatic nerves

**Figure 6:**
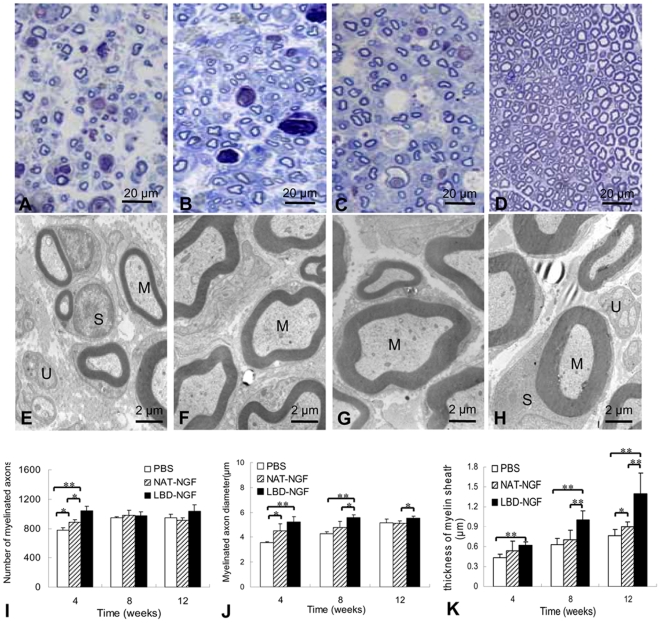
Remyelination of sciatic nerves. (a–d) Toluidine blue staining. At week 4 after injury, light micrographs of transverse semi-thin sections at the injury sites of the control (a), NAT-NGF (b) and LBD-NGF group (c) compared with that of native nerve group (d). (e–h) Transmission electron micrographs (TEMs). At week 12 after injury, ultra-thin sections at the injury sites of the control (a), NAT-NGF (b) and LBD-NGF group (c) compared with that of native nerve group (d) were observed under TEM. (i) The statistical analysis of the number of myelinated axons. (j) The statistical analysis of the myelinated axon diameter. (k) The statistical analysis of thickness of myelin sheath. Myelinated axons (M), unmyelinated axons (U) and Schwann cells (S) surrounding the myelinated axons can be seen clearly. n = 6, *, P<0.05, **, P<0.01, determined by two-tailed student's *t*-test.

Transverse semi-thin sections at the injury sites of sciatic nerves were analyzed by Toluidine blue staining. The results showed at week 4 after injury, a large number of well-myelinated axons with large diameter were observed in the group of LBD-NGF (Figure 6A–D).

Ultra-thin sections were observed under the transmission electron microscope. The results showed at week 12 after injury, the thickness of the myelin sheath around the axons was the greatest for native nerves, followed by LBD-NGF and then NAT-NGF treated nerves. The thickness of the myelin sheath in the control group was the thinnest (Figure 6E–H).

The statistical analysis revealed that at week 4 after injury, there were significant differences in the number of myelinated axons among the groups of the control, NAT-NGF and LBD-NGF (n = 6, P<0.05) (Figure 6I). However, at week 8 and 12, there was no significant difference among the three groups (n = 6, P>0.05) (Figure 6I).

In the statistical analysis of the myelinated axon diameter, we found at week 4, myelinated axon diameter was significantly larger in groups of NAT-NGF (n = 6, P<0.05) and LBD-NGF (n = 6, P<0.01) compared with the control (Figure 6J). But there was no significant difference between the groups of NAT-NGF and LBD-NGF (Figure 6J). At week 8 and 12 after injury, myelinated axon diameter of LBD-NGF group was significant larger than that of NAT-NGF (n = 6, P<0.05) (Figure 6J).

We also evaluated the thickness of myelin sheath by Image-Pro Plus software (Media Cybernetics). The statistical analysis revealed that at each time point, the myelin sheath thickness was significantly greater in the LBD-NGF group compared with the control (n = 6, P<0.01) (Figure 6K). At week 8 and 12, there were also significant differences in myelin sheath thickness between the groups of NAT-NGF and LBD-NGF (n = 6, P<0.01) (Figure 6K).

## Discussion

NGF plays a critical role in nerve injury repair. However in practice, NGF simply delivered in solution is difficult to be retained at the injury sites due to its rapid diffusion in extracellular fluids. In order to maintain NGF concentration, multiple injections are needed. However, they would increase cost and surgical risks. In addition, the excessive NGF at the injection sites and the diffusion of NGF may cause possible adverse effects. Thus, a delicate control of NGF both in dosage and in localization is critical to apply NGF safely and efficiently.

One of the strategies is to incorporate NGF into polymeric materials and then implant directly into the targeted tissue sites [22], [23]. It could maintain growth factor stability and prolong delivery of NGF, but the safety of this strategy remains to be a concern. In addition to polymer-based delivery systems, another approach to providing extended delivery of NGF is by using genetically-engineered cells, such as primary fibroblasts [24], polymer-encapsulated cell lines [25], or neural stem cell lines [26]. These strategies also allowed a prolonged delivery of NGF, but the safety of the method and the long-term NGF expression by transgene still need to be determined. Moreover, the delivered NGF doses that depend on the cell survival and the stability of the transfection could be difficult to control in a precise manner. At present, NGF delivery and cell transplantation could potentially be combined with other synthetic biodegradable scaffolds made of the poly (α-hydroxy acid) family of polymers [27] and of natural scaffolds such as laminin-fibronectin double coated collagen [28] used in nerve regeneration.

Laminin is widely dispersed in the peripheral nervous system [16], [17]. It has been shown that laminin-2 was a major matrix component of the PNS which could induce cell spreading and/or myelination [29]. It affects neuronal behavior, including proliferation, migration, target recognition, neurite outgrowth, and central synaptic differentiation [30], [31]. After peripheral nerve injuries, Schwann cells play an indispensable role in promoting regeneration by elaborating basement membrane containing extracellular matrix proteins, such as laminin [19]. Therefore, laminin could be a potential target for NGF which could be retained and enriched at the injury sites, enhance the efficacy of nerve regeneration.

Agrin is a key organizer of acetylcholine receptor (AChR) clustering at the neuromuscular junction. Binding of agrin to laminin requires NtA, which comprises the first 135 amino acids. This region mediates high-affinity interaction with the coiled-coil domain of laminin [21]. Thus, we fused NtA as a laminin-binding domain to NGF to specifically bind LBD-NGF to laminin. In order to validate its targeting effects, the laminin-binding activity of LBD-NGF was tested *in vitro*. As expected, LBD-NGF showed specific laminin-binding capacity compared with NAT-NGF which reflected by the lower Kd value. From the sustained release experiment, NAT-NGF was found to be quickly released at the first day, whereas LBD-NGF could gradually release from laminin during 6 days. These results demonstrated the LBD-NGF could be retained on laminin for a longer time compared with NAT-NGF.

We subsequently evaluated whether the bioactivity of NGF-β was influenced during the genetic engineering process. PC12 cells were used for examining the bioactivities of the refolded LBD-NGF by neurite outgrowth assay and MTT assay. The results showed that LBD-NGF retained the bioactivity of NGF. Bioactivity assay of NGF on laminin *in vitro* also demonstrated that LBD-NGF maintained higher concentration and bioactivities on laminin. The results suggested LBD-NGF could target to laminin and could be helpful in nerve injury repair.

The rat sciatic nerve crush injury model was used to test the effect of nerve injury repair by LBD-NGF. In order to limit additional variations, such as the surgical technique, mismatch of proximal and distal axonal alignment and foreign body (suture) reaction, we chose a pure crush injury model in the study. Sciatic nerve crush injuries caused anatomical disruption of axons. Wallerian degeneration occured when there was a disruption of the axon [32], [33]. The distal portion of the axon and myelin degenerated [34]. Therefore, this model provided a feasible system for studying the nerve regeneration, remyelination, and functional alterations associated with peripheral nerve injuries. In addition, this model partially maintained the continuity of the nerve. Thus, the nerve extracellular matrix component laminin could be utilized as the target as well as the scaffold for nerve regeneration. Functional assay by walking track analysis, histological analysis, and electrophysiological evaluation were the most commonly used assays to evaluate nerve regeneration in this model [35]–[37].

LBD-NGF was retained at the injury sites, and the LBD-NGF group demonstrated the most favorable functional recovery as measured by SFI. The electrophysiological observation of LBD-NGF group showed significantly improved restoration of NCV and DCMAP than NAT-NGF and the control groups correlated well with the functional SFI findings. In addition, the histological findings indicated that the LBD-NGF group showed better regeneration in axonal regeneration, Schwann cell proliferation and remyelination of sciatic nerves. Thus the *in vivo* work demonstrated that laminin binding NGF targeted specifically to the endogenous laminin of the sciatic nerves and maintained a higher concentration and stronger bioactivity of NGF at the injury sites. It was effective in enhancing histologically detectable nerve regeneration and improving functional recovery.

For nerve injuries such as crush and stretch, LBD-NGF could be directly utilized for repair by using the native nerve extracellular matrix component laminin as the target as well as the scaffold. However, if the nerve injury is extensive, forming an irreducible gap between the injured proximal and distal stumps, a nerve bridge technique would be preferred. Scaffolding biomaterials filled with laminin or laminin-rich biomaterials which can aid in the guidance of growing nerve fibers along appropriate paths could be used to enhance the precision of stump approximation. At the same time, the laminin binding NGF could be used to promote nerve regeneration and improve functional recovery. Laminin-rich nerve bridging scaffolds and the laminin binding NGF could be an effective strategy for the repair of severe nerve injuries.

In conclusion, LBD-NGF promoted sciatic nerve regeneration. LBD-NGF maintained a higher concentration and stronger bioactivity on laminin. Using a rat sciatic nerve crush injury model, LBD-NGF was found to be retained and concentrated at the nerve injury sites to enhance the functional restoration following nerve damages. The laminin binding NGF targeted to the nerve extracellular matrix laminin and promoted the repair of peripheral nerve injuries.

## Materials and Methods

### Engineering and preparation of NGF

Gene of NAT-NGF or LBD-NGF was generated by PCR and inserted into vector pET-28a (Novagen), respectively. BL21 (DE3) strain of *E. coli* was transformed targeted vector, and then we induced the protein of NAT-NGF or LBD-NGF with 1 mM isopropyl β-D-thiogalactopyranoside (IPTG) at 37°C for 5 h. we harvested the two recombinant proteins accumulating in the inclusion bodies from cell lysis by centrifugation and solubilized in 8 M urea containing 0.4% (vol/vol) β-Mercaptoethanol. Purification of the solubilized fusion proteins was then accomplished under denaturing conditions by nickel chelate chromatography (Amersham Biosciences). The expression of recombinant proteins was analyzed by electrophoresis on a 15% (wt/vol) sodium dodecyl sulfate polyacrylamide gel electrophoresis (SDS-PAGE) under reducing condition and western-blotting with mouse antibody to polyhistidine (1∶1,000 dilution; Sigma) and sheep antibody to mouse Alkaline phosphatase (1∶10,000 dilution; Sigma). The purified NAT-NGF or LBD-NGF was refolded in the glutathione redox-refolding system by chromatographic method [38], then stored at −80°C. Protein concentration was monitored by the method of Bradford utilizing bovine serum albumin (BSA) as a standard [39].

### Laminin binding assay *in vitro*

The laminin binding assay was carried out by a modified ELISA assay [40], [41]. We added laminin (Sigma) previously to 96-well plates (Costar), and then incubated the plates at 4°C for 24 h and ventilated dry after discarding the redundant solution, followed by washing for 3 times with PBS (pH 7.3). we blocked the plates with 2.5% (wt/vol) BSA containing 0.1% (vol/vol) Tween 20 at 37°C for 2 h. Recombinant proteins with increasing concentrations were added to the plates (100 µl/well) and incubated at 37°C for 2 h, and then washed for 3 times to remove the unbound proteins. Mouse monoclonal antibody to polyhistidine (1∶1,000 dilution; Sigma) was added and incubated at 37°C for 1 h. The unbound primary antibodies were removed by three washes as above. Sheep antibody to mouse Alkaline phosphatase (1∶10,000 dilution; Sigma) was added to incubate at 37°C for 1 h. After three washes as above, bound proteins were detected by the alkaline phosphatase (AP) reaction with *para*-nitrophenylphosphate (*p*NPP) (Sigma) in AP buffer (pH 9.6) for 15 min at 37°C. The results were quantified at 405 nm using an ELISA reader (Molecular Devices). We calculated the dissociation constant Kd values for two proteins binding to laminin as described previously[42].

### Sustained release assay of NGF from laminin *in vitro*

In this study, we prepared the laminin containing membranes from pig amnion [43], [44]. We tested the laminin content of the scaffold by ELASA assay. Antibody to laminin (1∶1,000 dilution; Abcam) was used in this experiment. Laminin (20 µg/ml) purchased from Sigma-Aldrich. Inc was used as the positive control. Then the laminin membranes (6-mm diameter) were loaded with 0.05 nmol NAT-NGF or LBD-NGF respectively, and placed in 48-well plates. The membranes were suspended in 500 µL PBS and incubated on a rocker platform (37°C, 80 g). PBS in 48-well plates was changed every 12 h. At each time point from day 0 to 8, we collected the samples and analyzed the NAT-NGF and LBD-NGF retained on the membranes by ELISA assay.

### Bioactivity assay of NGF *in vitro*

We measured the bioactivity of NAT-NGF and LBD-NGF using PC12 cells (purchased from the Peking Union Medical College) in cell neurite outgrowth assay [45], [46] and MTT assay [47]. PC12 cells were seeded in polylysine-treated 48-well plates (Costar) at the density of 5×10^3^ cells/well and maintained under serum-free RPMI 1640 medium (HyClone) at 37°C for 1 h, then serially diluted refolded NAT-NGF or LBD-NGF was added to the plates. After 24 h incubation, the percentage of cells with neurites more than the average diameter of the cell in length was determined. The number of surviving cells was determined by MTT assay after 72 h culture with NAT-NGF or LBD-NGF. Cells cultured under identical conditions but without NGF served as a blank control. The assay was valued in duplicate by two independent assessors.

### Bioactivity assay of NGF on laminin *in vitro*

In this study, laminin-coated 48-well plates (Costar) were incubated with NAT-NGF or LBD-NGF at 4°C for 1 h, and then the plates were extensively washed. PC12 cells were seeded at the density of 5×10^3^ cells/well under the same condition as described above. After 24 h incubation, the percentage of cells with neurites more than the average diameter of the cell in length was determined. The number of surviving cells was also determined by MTT assay after 72 h culture. Cells cultured under identical conditions but without NGF served as a blank control. The assay was valued in duplicate by two independent assessors.

### Laminin content evaluation in rat sciatic nerve

Six rats were sacrificed by cervical dislocation. The sciatic nerves about 1 cm of 3 rats were rapidly excised and fixed in 4% (vol/vol) formaldehyde for 48 h. we embedded the samples in paraffin and 5-µm sections and examined the samples by immunohistochemistry using antibody to laminin (1∶2,000 dilution; Chemicon). The sciatic nerves about 1 cm of the other 3 rats were also excised, frozen immediately in liquid nitrogen. Then we extracted the proteins for western-blotting analysis. In the analysis, we used the antibody to laminin (1∶2,000 dilution; Chemicon) to test the laminin content in the rat sciatic nerve. At the same time, we used laminin (10 µg) purchased from Sigma-Aldrich.Inc as the positive control.

### Surgical Procedures and application of NGF

To test the laminin binding ability and biological function of NAT-NGF and LBD-NGF *in vivo*, we used a rat sciatic nerve crush injury model. Seventy-eight male Sprague-Dawley rats weighting 200–220 g were anesthetized by an intraperitoneal injection of sodium pentobarbital (40 mg/kg body weight) and the hair on the right femur was removed. After sterilization, subcutaneously an incision was made at the thigh. Right sciatic nerve was carefully exposed from the intermuscular space, isolated, and subsequently crushed a 5 mm segment for 10 s above the bifurcation of the sciatic nerve with a hemostatic forceps. Then we used a non-degradable suture to mark the site of the lesion at the epineurium. 10 µl NAT-NGF (30 µM) or LBD-NGF (30 µM) was injected into the injury sites respectively. We injected PBS as negative control. Finally, we sutured the fascia and skin separately. During these animal experiments, we observed the Chinese Ministry of Public Health (CMPH) Guide and US National Institute of Health (NIH) Guide for the care and use of laboratory animals. All animals were kept under standardized laboratory conditions in an air-conditioned room with free access to food and water. At different time points, the rats were sacrificed by cervical dislocation.

### Laminin binding assay of NGF *in vivo*

Three hours and twelve hours after injection of NAT-NGF or LBD-NGF, 24 rats were sacrificed by cervical dislocation. We excised the injured sciatic nerves about 1 cm, frozen them immediately in liquid nitrogen. Then we extracted proteins for western blot analysis. Antibody to polyhistidine (1∶1,000 dilution; Sigma) was used to distinguish exogenous proteins from endogenous proteins.

### Walking track analysis

We assessed the functional nerve recovery following sciatic nerve injury using rat walking track analysis by recording its footprints. At days 0, 7, 11, 14, 18, 21, 25, 28, 35, 42, 49, 56, 70 after nerve injury the animals were submitted to walking track analysis and measurement of the SFI using a method described by Bain et al [37]. We could calculate the SFI based on the following formula:
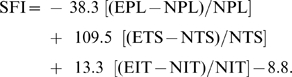



Where EPL indicated the operated experimental paw length; NPL, normal paw length; ETS, operated experimental toe spread, i.e., the distance between the first and fifth toes; NTS, the normal toe spread; EIT, the operated experimental intermediary toe spread, i.e., the distance between the second and fourth toes; and NIT, normal intermediary toe spread. The SFI was scaled such that −100 represented the sciatic nerve was crushed completely and 0 represented normal function or completely recovery [48]. The evaluations of all animals were performed by a single investigator blinded to the experiments.

### Electrophysiological evaluation

We performed the electrophysiological evaluation before, immediately after, and weeks 4, 8, 12 after the sciatic nerve injury. In this experiment we evaluated two parameters, NCV and DCMAP. Under anesthesia, the rats' sciatic nerves were exposed. Monopolar recording electrode and bipolar stimulating electrodes were used to induce and record electrical activity. We recorded NCV and DCMAP in responding to the stimuli using EMG recorder (Medelec Synergy, Oxford Instruments). The following settings were used: Low Filter, 3 Hz; High Filter, 10 KHz; Sensitivity, 0.1 mV; Sweep duration, 10 ms.

### Histological analysis

We performed the morphological assessments at weeks 4, 8 and 12 after sciatic nerve injury. The sciatic nerves (about 1 cm) at the injury sites were rapidly excised and fixed in 4% (vol/vol) formaldehyde for 48 h. The segments were embedded in paraffin and 5-µm sections were cut from each segment examined by H.E. staining and immunohistochemistry using antibodies to neurofilament (1∶100 dilution; Thermo) and S100 (1∶2,000 dilution; Sigma). Using Image-Pro Plus software (Media Cybernetics), we performed the quantification of neurofilament-positive and S100-positive area from at least 6 randomly selected fields at 400× magnification.

We stained the transverse semi-thin sections (1 µm thick) from each segment with Toluidine blue and observed under a light microscope (performed by Jinan Lujing Microscopic Technical Center). The number of myelinated axons was counted from at least 6 randomly selected fields under the magnification of ×400. Using Image-Pro Plus software (Media Cybernetics), we evaluated the myelinated axon diameter at 400× magnification. Ultra-thin sections (70 nm thick) were observed under transmission electron microscope (performed by Jinan Lujing Microscopic Technical Center). We also evaluated the thickness of myelin sheath by Image-Pro Plus software (Media Cybernetics).

### Statistical analysis

Data were presented as mean values±standard deviation. The statistical significance of differences in parameters was determined as *P<0.05 and **P<0.01, by two tailed student's t-test.
